# A clash on the Toll pathway: competitive action between pesticides and zymosan A on components of innate immunity in *Apis mellifera*

**DOI:** 10.3389/fimmu.2023.1247582

**Published:** 2023-09-11

**Authors:** Dani Sukkar, Ali Kanso, Philippe Laval-Gilly, Jairo Falla-Angel

**Affiliations:** ^1^ Université de Lorraine, INRAE, LSE, F-54000 Nancy, France; ^2^ Biology Department, Faculty of Sciences I, Lebanese University, Hadath, Lebanon

**Keywords:** Toll pathway, immune modulation, β-glucan, imidacloprid, amitraz, honeybees, immune genes

## Abstract

**Background:**

The immune system of honeybees includes multiple pathways that may be affected by pesticide exposure decreasing the immune competencies of bees and increasing their susceptibility to diseases like the fungal Nosema spp. infection, which is detected in collapsed colonies.

**Methods:**

To better understand the effect of the co-presence of multiple pesticides that interact with bees like imidacloprid and amitraz, we evaluated the expression of immune-related genes in honeybee hemocytes.

**Results:**

Imidacloprid, amitraz, and the immune activator, zymosan A, mainly affect the gene expression in the Toll pathway.

**Discussion:**

Imidacloprid, amitraz, and zymosan A have a synergistic or an antagonistic relationship on gene expression depending on the level of immune signaling. The presence of multiple risk factors like pesticides and pathogens requires the assessment of their complex interaction, which has differential effects on the innate immunity of honeybees as seen in this study.

## Graphical Abstract

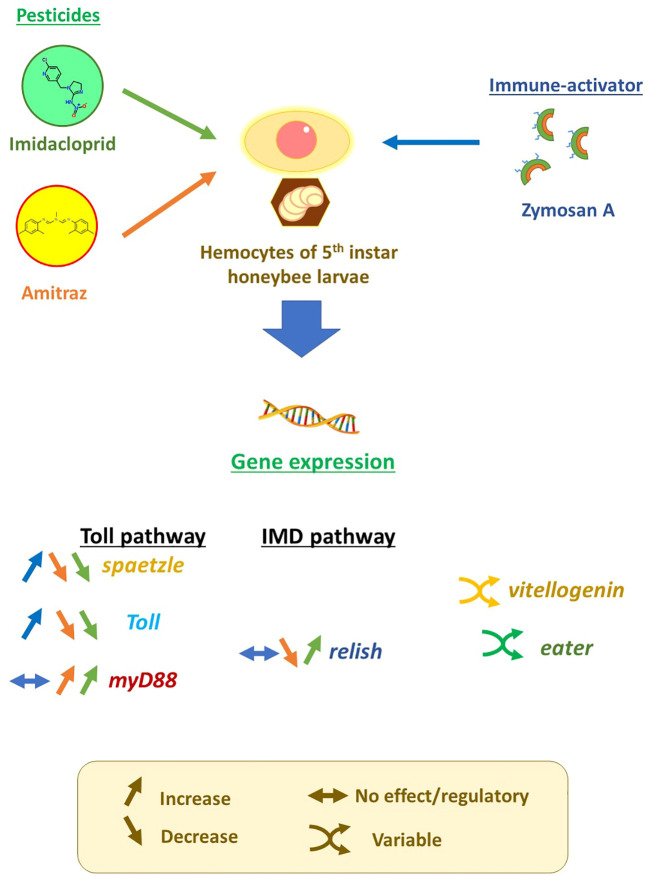

## 1 Introduction

Pesticide application is involved in the decline of insects and generally arthropods worldwide. Simultaneously, honeybees (*Apis mellifera*) are facing a global phenomenon termed colony collapse disorder (CCD) characterized by the disappearance of worker bees leaving the queen and brood unattended leading to colony death (1, 2). Studies were not able to pinpoint the exact cause of this phenomenon but the concurrence of multiple risk factors including pathogens and pesticides may be implicated (3). However, the microsporidian *Nosema* spp. are unicellular parasites that infect honeybees (4). *Nosema* spp. were found to be a potential contributing factor to colony collapse with prevalence in CCD colonies (5, 6).

Pathogenesis may increase when bees are exposed to pesticides shedding light on the effect of the interplay of different risk factors leading to CCD. Any alteration of the immune pathways may affect the organism’s ability to fend off pathogens and diseases. In fact, imidacloprid was found to decrease the expression of immune-related genes in honeybees (7) and increased *Nosema* spore production was also observable in bees exposed to imidacloprid (8). The exposure to *N. ceranae* and the neonicotinoid, thiamethoxam, resulted in dysbiosis of honeybee gut microbiota (9). Other studies that considered pesticide co-exposure with Nosema found alteration in gut microbiota as well (10, 11). This strongly suggests a relationship between pesticide and pathogen exposure and the synergism of their interaction. In addition, *Nosema* infection alters honeybee mitochondrial metabolism exerting stress, and the infection leads to a decrease of antimicrobial peptide production (12).

In the context of bee immunity, imidacloprid is a pesticide that gives rise to great concern not just because of its effect on increased susceptibility to diseases, but also because it is the world’s most-used neonicotinoid pesticide (13). Imidacloprid also has the highest percentage of neonicotinoid residues in honey in most continents (14). However, imidacloprid is not the only pesticide of concern in beekeeping. Amitraz is an acaricide used to treat *Varroa* infections in bee colonies (15) with direct interaction with honeybee colonies and the most common pesticide found in honey (16). Although amitraz was deemed safe for honeybees since bees have low hydrolysis of amitraz to its active metabolites (17), amitraz appears to amplify the effect of other pesticides like tau-fluvalinate and coumaphos, while the effect of amitraz itself remains unchanged regarding lethality to honeybees (18). In addition to its synergistic effect with co- presence with other pesticides, amitraz exposure resulted in an increased titer of the Israeli acute paralysis virus (IAPV) in bees exposed to amitraz (19), indicating the role of amitraz in increasing disease susceptibility.

**Figure 1:**
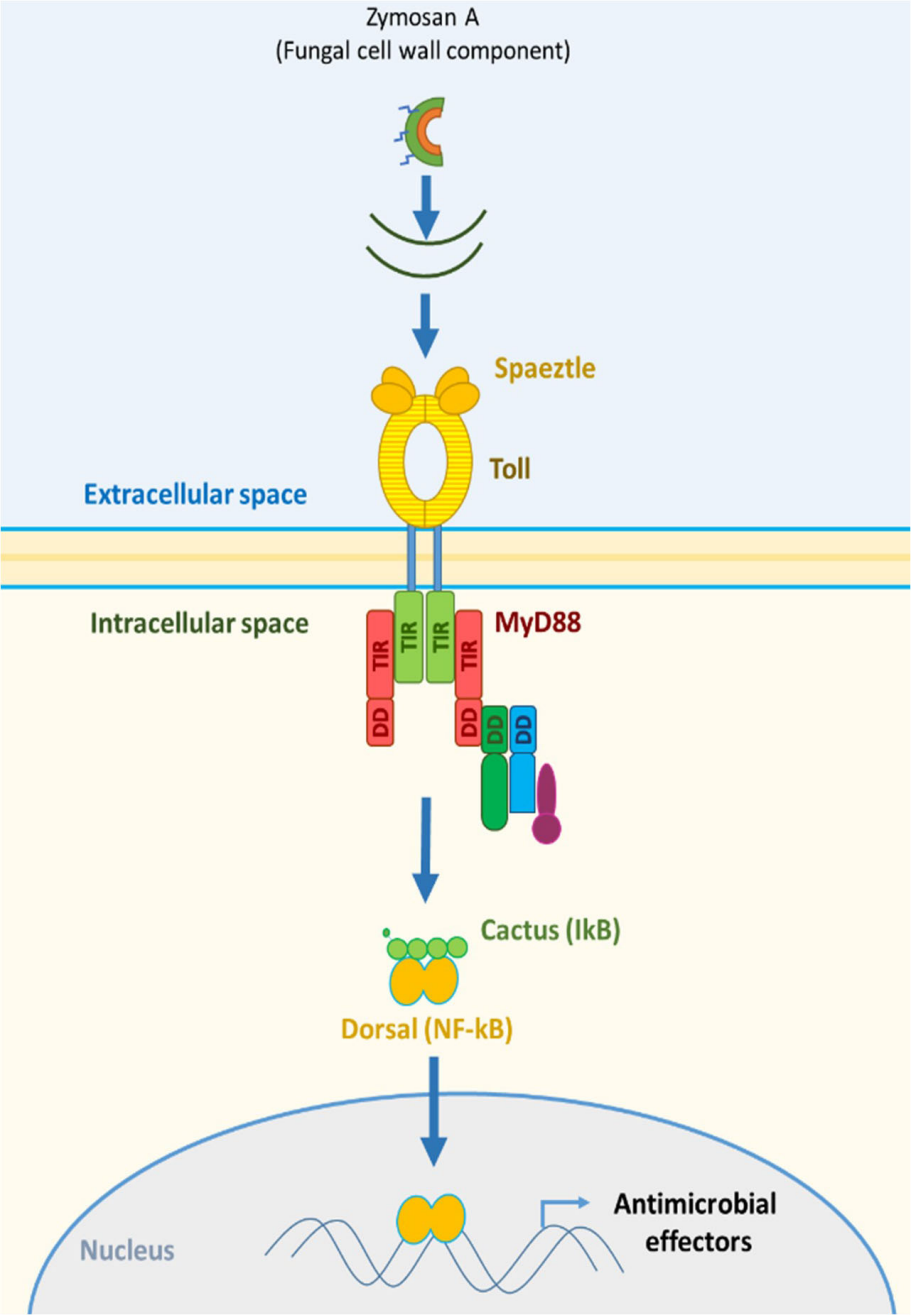
Response to zymosan A exposure in the Toll pathway of the invertebrate immune system.

Increased pathogen susceptibility when exposed to pesticides refers to the alteration of the insect immune system as the primary point of investigation. The insect immune system relies on the innate immune system to fend off pathogens and diseases if physical barriers are breached (20, 21). Phagocytosis is one the most important mechanisms to fend off infectious agents in an organism that relies on innate immunity and lacks the same complexity as the vertebrate immune system. Eater and NimC1 are phagocytic receptors termed NIMs (Nimrods) and are main components in phagocytosis (22). Eater, in particular, is involved in the uptake of Gram-positive bacteria by direct binding (23). Eater is also involved in the mobility and adhesion of hemocytes (24); thus, it may affect the response to pathogenic infections. Eater appears to compensate for the function of NimC1 in *NimC1* null *Drosophila* mutants, implying the crucial need for Eater activity in phagocytosis. After the discovery of the Toll immune signaling pathway and the Toll receptors in *Drosophila*, the Toll pathway became a focus in innate immunity. The activation of the Toll pathway is implicated in elucidating innate immunity in vertebrates and invertebrates (25–28). In honeybees, the activation of the Toll pathway results in the production of antimicrobial peptides as a protective response to pathogenesis (29). Bacterial and fungal recognition is mediated by the interactions of pathogen-associated molecular patterns (PAMPs) with recognition proteins that result in the activation of a cytosolic receptor, Spaetzle (Spätzle). PAMPs include cell wall components of Gram-positive bacteria (peptidoglycans), Gram-negative bacteria (lipopolysaccharides), and fungi (β-glucans) like zymosan (29–33). Spaetzle is important in the first steps of pathogen recognition leading to the activation of the designative Toll receptor of the Toll pathway (34) (**Figure 1**).

This activation, in turn, leads to signal transduction via various proteins including the myeloid differentiation factor 88 (MyD88), which is a key modulator in the Toll pathway (35) and was reported to be crucial in the immune response in mice (36). The role of MyD88 extends to phagocyte maturation and its implications in phagocytosis (37). Zymosan A poses a validated activator of the Toll pathway (38–41). Regarding other immune pathways, Relish is also a key intracellular modulator in the IMD signaling pathways and mediated a cross-talk between IMD and JNK pathways inferring a strong role in bee immunity (29). However, honeybee immunity is not limited to direct elements of immune pathways but also to elements that are implicated in a form of social immunity like the hemolymph protein vitellogenin (42). Vitellogenin mediates trans-generational immune priming (TGIP) (43, 44), i.e., the transfer of immune experience from adult bees to larvae. Vitellogenin is also connected to aging in honeybees by its antioxidant activity (45). All the mentioned components may contribute greatly to understanding the impact of pesticides and their combinations on honeybee immunity in a comprehensive manner.

In this study, we aim to evaluate the effect of pesticides and their different combinations on the modulation of genes implicated in immune responses in honeybees to better understand the toll of pesticide exposure on the fitness of honeybees and the potential cause of disease susceptibility. This is done by applying imidacloprid and amitraz to hemocytes challenged with different concentrations of the fungus-derived immune stimulator, zymosan A, to mimic interaction with *Nosema* spp.

## 2 Materials and methods

### 2.1 Hemolymph extraction

Hemolymph was extracted from European honeybee (*A. mellifera*) larvae at the fifth stage of development. Extraction was done under a laminar flow hood by sterilizing the abdominal dorsal section with 70% ethanol. The segment is then punctured with a sterile needle allowing the outflow of hemolymph that was rapidly collected by a micropipette and pooled in WH2 medium, which was prepared as described by Hunter (46) and filtered through a 0. 2-µm syringe filter (Acrodisk™ 4312, Pall corp. ™). Fifty larvae were pooled for each 2 ml of WH2 medium used in batches.

### 2.2 Pesticides and zymosan exposures

Imidacloprid (37894-100 mg, Sigma-Aldrich™) and amitraz (45323, Sigma-Aldrich™) stock solutions were dissolved in WH2 medium, each to two concentrations, 40 and 200 µg/ml, while zymosan A (Z4250, Sigma-Aldrich™) stock solution had a concentration of 4 µg/ml. All stock solutions were sonicated for 30 min in a water-bath sonicator before usage to ensure dissolution of the pesticides and zymosan A. Treatments and/or WH2 medium were added to 24-well tissue culture plates (92024, TPP™) containing 100 µl of diluted hemolymph to reach a total volume of 400 µl. In addition to the treatment control, the final concentrations of treatments were 10 or 50 µg/ml for either imidacloprid or amitraz single exposures. Pesticide mixtures were set to the final concentrations of 10 + 10 µg/ml, 10 + 50 µg/ml, or 50 + 10 µg/ml of imidacloprid and amitraz. All pesticide treatments were done in either no zymosan or with 1 µg/ml zymosan. Plates were sealed with sterilized sealing tape and incubated at 20°C in the dark for 18 h. All conditions were made in triplicates (*n* = 3).

### 2.3 RNA extraction

The incubation was terminated and the supernatant was transferred from each well to corresponding Eppendorf tubes that were centrifuged at 5,000 rcf for 5 min. TRIzol reagent (400 µl; 15596018, Thermofisher™) was added to each well following the user protocol (MAN0001271) for RNA extraction. The supernatant was removed from the centrifuged tubes and the lysates were pipetted several times in the wells before transferring them to their corresponding tubes. The tubes were frozen at −80° C before the continuation of the extraction. After thawing, an 80-µl volume of chloroform was added to each tube, inverted several times, and incubated for 3 min followed by centrifugation at 12,000 rcf for 15 min at 4°C. Most of the upper phase was transferred to a new tube and 400 µl of isopropanol was added. The samples were incubated for 10 min at 4° C then centrifuged at 12,000 rcf for 10 min at 4° C. The supernatant was discarded. Ethanol (75%) was added to each sample in 400 µl volume and vortexed briefly. Tubes were then centrifuged at 7,500 rcf for 5 min at 4° C. Supernatant was discarded and each sample was suspended in 40 µl in RNase-free water. Sample concentration and purity were quantified by BioSpecNano spectrophotometer (Shimadzu corps™).

### 2.4 cDNA synthesis

RNA of each sample was reverse transcribed to cDNA using a RevertAid H Minus First Strand cDNA Synthesis Kit (K1632, Thermofisher™). Tubes were placed on ice blocks and 500 ng of RNA of each sample was added to a PCR tube followed by the addition of 1 µl of oligo (dT)_18_ primer, 4 µl of 5× reaction buffer, 1 µl of RiboLock RNase inhibitor (20 U/µl), 2 µl of 10 mM dNTP mix, and 1 µl of RevertAid H minus M-Mul V reverse transcriptase (200 U/µl). RNase-free ultra-pure water was added to reach 20 µl total volume per tube. Sample tubes were incubated in an ICycler thermocycler (Bio-Rad™) at 60°C for 42 min and then heated to 70° C for 5 min. The samples were held at 4°C before removal from the thermocycler. Sample concentration and purity were quantified by BioSpec Nano spectrophotometer (Shimadzu corps™). Samples were diluted by a factor of 10 and stored at −80°C until usage.

### 2.5 Melting temperature (Tm) gradient

A temperature gradient analysis was set for honeybee primers between 50 and 63°C. Analysis was performed with Hard-Shell High-Profile Semi-Skirted 96-Well PCR Plates (Bio-Rad™). Each well contained 10 µl of SsoAdvanced™ Universal SYBR^®^ Green Supermix (#172-5271, Bio-Rad™), 2 µl of honeybee larvae cDNA, 0.5 µM of forward and reverse primers (final concentration), and ultra-pure H_2_O added until 20 µl total volume. Forward and reverse primer sequences were chosen for *spaetzle, relish, toll, myD88, eater, vg (vitellogenin)*, and *rp49* genes as indicated in **Table 1**.

**Table 1:** Primer sequences for honeybee gene real-time PCR.

Gene target	Forward primer	Reverse primer	Gene ID	Source
***spaetzle***	5’-TGCACAAATTGTTTTTCCTGA-3’	5’-GTCGTCCATGAAATCGATCC-3’	GB15688	(29)
***relish***	5-GCAGTGTTGAAGGAGCTGAA-3’	5-CCAATTCTGAAAAGCGTCCA-3	GB13742	(29)
***toll***	5’-TAGAGTGGCGCATTGTCAAG-3’	5’-ATCGCAATTTGTCCCAAAAC-3’	GB18520	(29)
***myD88***	5’-TCACATCCAGATCCAACTGC-3’	5’-CAGCTGACGTTTGAGATTTTTG-3’	GB12344	(29)
***eater***	5’-CATTTGCCAACCTGTTTGT-3’	5’-ATCCATTGGTGCAATTTGG-3’	XP_001120277	(47)
***vg (vitellogenin)***	5’-AGTTCCGACCGACGACGA-3’	5’-TTCCCTCCCACGGAGTCC-3	NP_001011578	(47)
***rp49***	5’-CGTCATATGTTGCCAACTGGT-3’	5’-TTGAGCACGTTCAACAATGG-3’	AF441189	(48)

### 2.6 Real-time polymerase chain reaction

Gene expression analysis of for *spätzle, relish, toll, myD88, eater*, and *vg* was performed by iCycler MyiQ™2 Two-color Real-Time Detection System (Bio-Rad™) in Hard-Shell High-Profile Semi-skirted 96-Well PCR Plates with *rp49* as a housekeeping gene. Reaction mixtures contained 10 µl of SsoAdvanced™ Universal SYBR^®^ Green Supermix, 0.5 µM of forward and reverse primers (final concentration), and 300 ng of cDNA, and ultra-pure H_2_O was added to a total volume of 20 µl.

Reaction cycles set: 1× (30 s at 95°C); 45× (10 s at 95°C, 30 s at 58°C, 30 s at 72°C) followed by melt curve analysis increasing temperature from 55 to 95°C. Each sample was analyzed with two technical replicates to check for repeatability.

### 2.7 Statistical analysis

Statistical analysis was performed using Addinsoft^®^ XlSTAT™ 20198.3.2. Data were checked for normality by a Shapiro–Wilk test, and homogeneity of variance was tested by Bartlett’s test. Non-normal data were transformed and normalized before performing a two-way ANOVA coupled with a Duncan *post-hoc* to determine significant differences between groups. A principal component analysis (PCA) and factor map analysis were carried out to determine the correlation between the expression of different genes. The correlation was checked for each individual treatment within zymosan groups or for the whole groups by Pearson (*n*) test at a 95% confidence interval (*n* = 3).

## 3 Results

**Figure 2:**
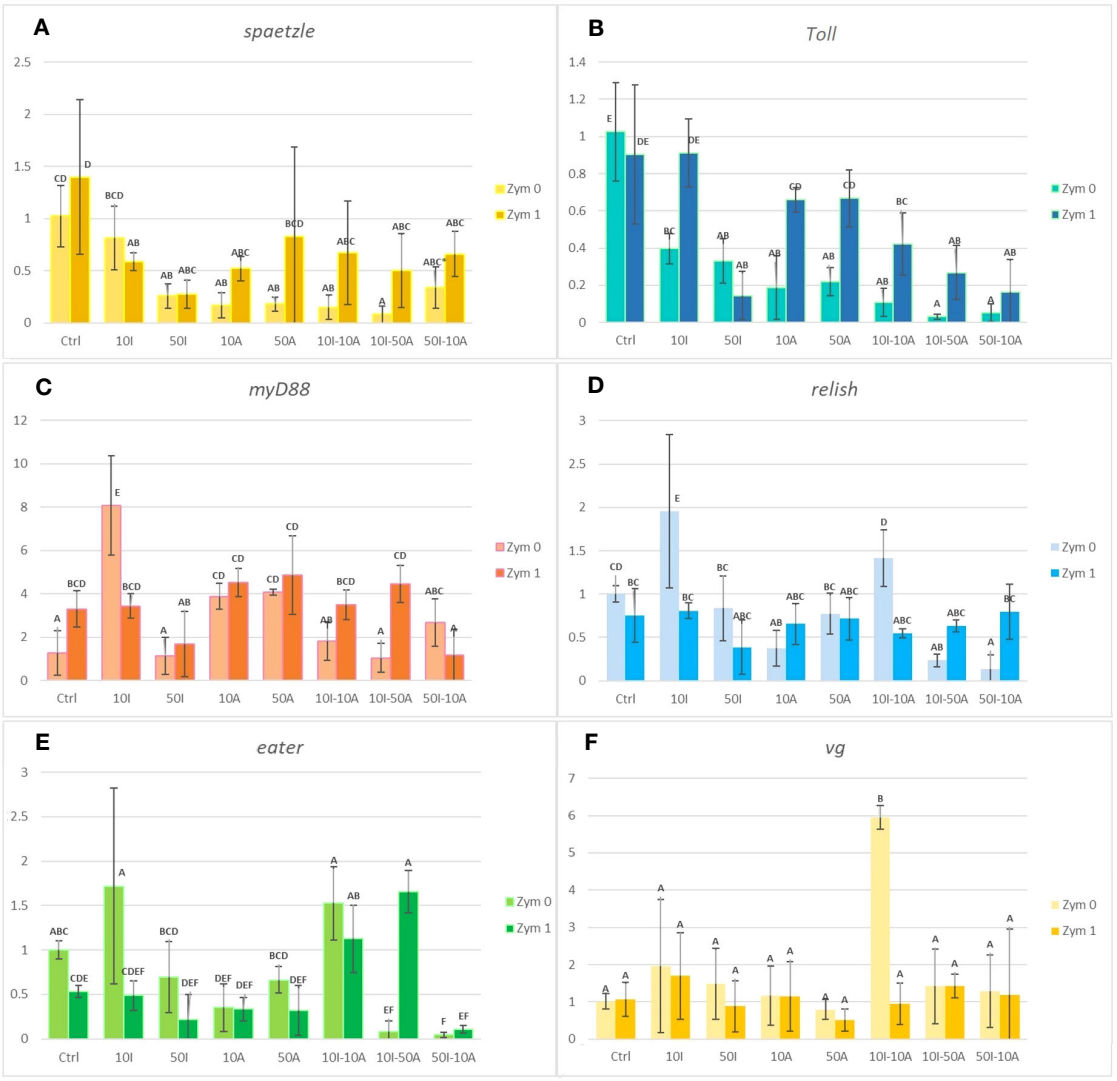
Immunity- and development-related gene expression analysis in *Apis mellifera*. Hemocytes exposed to 10 or 50 µg/ml imidacloprid (10I or 50I) or amitraz (10A or 50A) either as separate exposures or as combinations (10I-10A, 10I-50A, and 50I-10A). All pesticide treatments were done without immune challenge by zymosan (Zym 0) or with 1 µg/ml zymosan (Zym 1). Different letters indicate significant differences (*n* = 3, confidence interval = 95%). Error bars represent standard errors (SE). Graphs **(A-F)** represent the expression of *spaetzle, Toll, myD88, relish, eater*, and *vg*, respectively.

The gene expression of *spaetzle* is represented in **Figure 2A**. All pesticide exposures of imidacloprid and amitraz decreased the expression of *spaeztle* whether in single exposures or in co-exposures. This is also true for both groups of zymosan exposition (Zym 0 and Zym 1). In Zym 0, the 10I treatment was not significantly different from the control but 50I, 10A, 10I-10A, and 10-50A showed a significant decrease compared to the control of the group with 10I-50A having the most decrease. The 50I-10A mixture is only significantly different from the control with mean difference considered only within the same zymosan group and not all the treatments. The same effect is observed with a significant decrease in gene expression in all treatments with 1 µg/ml zymosan A compared to the control with the exception of the 10 µg/ml imidacloprid treatment. Comparing each treatment with correspondence to its Zym 0 and Zym 1 though does not show statistical difference; it is still observable that, with zymosan A, the expression of *spaetzle* increases in all treatments except the single exposures with imidacloprid.

Imidacloprid and amitraz significantly lowered the expression of *Toll* in all treatments when not immunologically challenged by zymosan A (**Figure 2B**). In fact, *Toll* expression decreased by at least 60% compared to the control when exposed to 10 µg/ml imidacloprid. A significant decrease is observed in single exposures, but the impact is even more significant in double-pesticide exposures especially in mixtures that have 50 µg/ml of either imidacloprid or amitraz. In the presence of zymosan A, there was no effect of 10 µg/ml imidacloprid or amitraz single exposures on *Toll* expression compared to the zymosan control. The decrease was significantly present with the pesticide mixtures and 50 µg/ml imidacloprid.

When we compare treatments between the absence and presence of zymosan A (Zym 0 and Zym 1 respectively), we can observe that the effect of 10 µg/ml imidacloprid and both single concentrations of amitraz is not significantly different from the Zym 1 control. In addition, zymosan did not induce the over-expression of *Toll* when we compare the control of Zym 0 and Zym 1 groups.

Comparing the two controls with and without zymosan, the MyD88 expression significantly increases when hemocytes are treated with zymosan (**Figure 2C**). When not exposed to zymosan, *myD88* expression remains the same with the imidacloprid and amitraz mixtures in addition to the 50 µg/ml imidacloprid treatment. However, both amitraz exposures significantly increased the expression compared to the control and the 10 µg/ml imidacloprid exposure even increased the expression further and more significantly than the amitraz exposure.

Hemocytes challenged with zymosan A have no significant change in *myD88* expression except for the 50I-10A mixture, which was significantly lower than all treatments in the Zym 1 group including the zymosan control except for the treatment of 50 µg/ml imidacloprid. The 10I treatment is significantly higher than its corresponding treatment with zymosan. The case is inverted in most other treatments where pesticide treatments that included zymosan were higher in terms of *myD88* expression than without zymosan. However, the effect of zymosan in a significant increase is only observed in the control and the 10I-50A treatment.

Similar to the effect on *relish* and *myD88* when hemocytes were not exposed to zymosan, 10 µg/ml imidacloprid resulted in an increase in the expression of the *eater* gene implicated in phagocytosis (**Figure 2E**). A significant decrease was observed with the mixtures 10I-50A and 50I-10A compared to the Zym 0 control. As for the pesticide treatments that included zymosan exposure, no significant change was observed regarding expression of *eater* except with the 10I-50A mixture compared to the Zym 1 control. In addition, when comparing treatments with and without zymosan, we only observe a significant decrease with 10I when treated with zymosan and the inverse in the 10I-50A treatment. Amitraz may antagonize the effect of imidacloprid when it is either at the same concentration as seen with *relish* expression or when amitraz concentration exceeds that of imidacloprid as seen with eater expression in the context of immune activation.

In **Figure 2D**, pesticide treatments that included 10 µg/ml imidacloprid either alone or in a mixture with amitraz induced a significant increase in the expression of *relish* when hemocytes are not exposed to zymosan. A significant decrease in gene expression was observed with 10A, 10I-50A, and 50I-10A treatments without zymosan.

We can observe that there is no significant difference in *eater* expression between treatments when comparing in the absence and presence of zymosan except for 10I and 10I-50A, which are significantly lower and higher after immune activation, respectively (**Figure 2E**). With zymosan A, the expression of *eater* was not significantly different in single exposures and the 50I-10A mixture compared to the control. Intriguingly, the 10I-10A and 10I-50A treatments showed a significant increase when compared to the control with zymosan exposure. Furthermore, the 10I-50A treatment illustrated a contrasting result on gene expression with or without zymosan A. When not immunologically challenged, the eater gene expression with 10-50A treatment decreases significantly but increases significantly when zymosan is included.

Imidacloprid and amitraz appear to have no effect on the production of *vitellogenin (vg)* gene regardless of exposure to zymosan with the exception of 10I-10A (**Figure 2F**). This peculiar case infers that at given concentrations, imidacloprid and amitraz may act as reproductive disruptors when present simultaneously but not with single exposure or with mixtures of higher concentrations. A regulatory response may be activated to prevent alteration in *vg* levels. This could be connected to the observed trade-off between phagocytosis and the cytoprotective response. However, oral administration of sub-lethal doses of imidacloprid decreased vitellogenin expression levels in honeybees (49), suggesting that if there is an effect on vitellogenin from combinations and imidacloprid or amitraz, it may be more visible at the level of the whole organism or other types of cells. Variable degrees of vitellogenin expression were also reported between caged bees and bees in the field when exposed to 5 and 200 ppb imidacloprid from 1 to 2 days (50).

As for *vitellogenin (vg)*, there was no change in gene expression in any pesticide treatment despite the presence or absence of zymosan (**Figure 2F**). The exception is the pesticide mixture of 10I-10A where the expression of vg was significantly higher than all other treatments.

**Figure 3:**
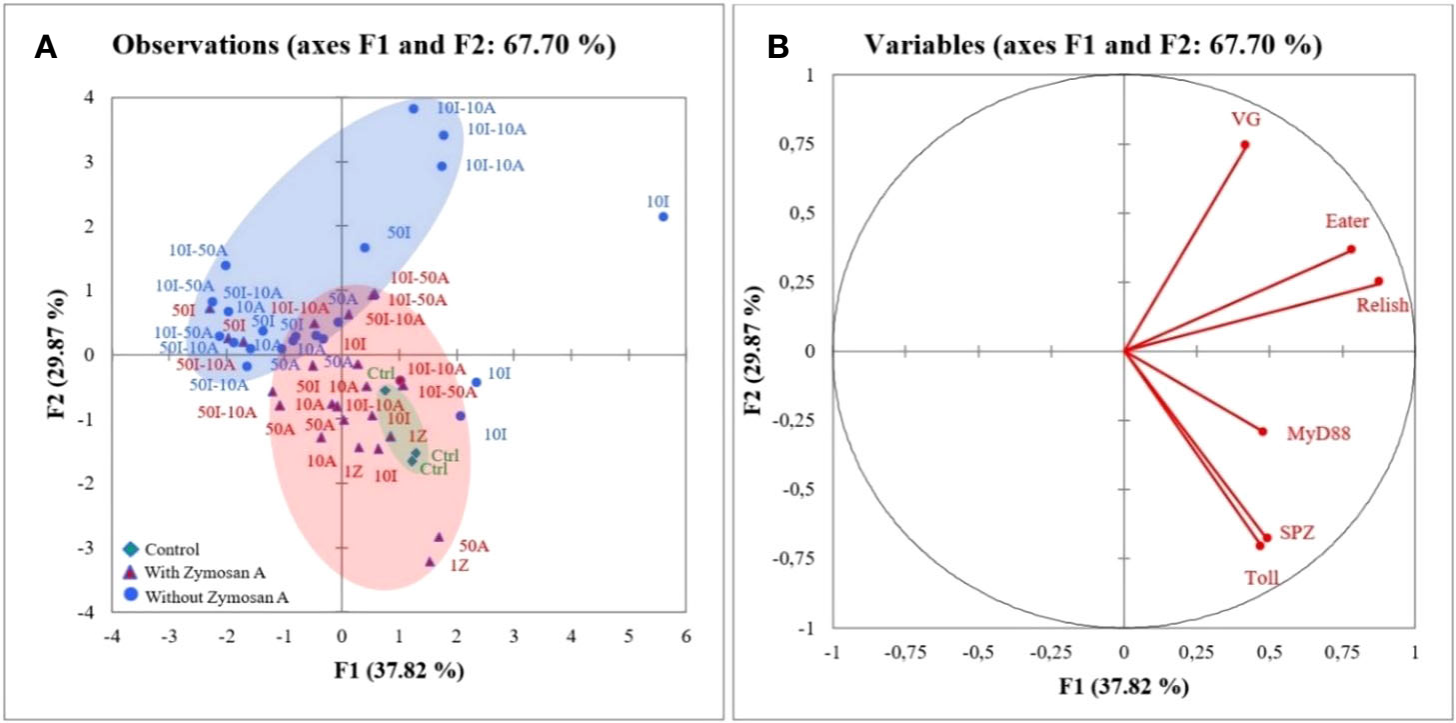
Principal component analysis (PCA) generated from expression of genes after zymosan A application in honeybee hemocytes exposed to different concentrations of imidacloprid and amitraz. **(A)** Individuals’ factor map according to the treatment (*n* = 3). Individuals are colored according to their membership in the modalities of the variables. **(B)** Factor map of gene expression involved in the discrimination of different treatments.

In **Figure 3**, the first two axes of PCA express 67.7% of the total inertia of variability of the obtained data. The graph of individuals (**Figure 3A**) on the factorial design shows that axis 1 pits the treatment without zymosan A application against the control and the honeybees treated with zymosan A. This axis 1 explains 37.8% of the variation. The separation of two groups on this axis revealed that control and honeybee hemocytes treated with zymosan A were positively correlated to *myD88*, *spaetzle*, and *Toll* gene expression. Regarding the representation of the variables on the factorial plane (**Figure 3B**), axis 1 shows groups of strongly contributing variables. The variables *eater, relish*, and *myD88* are characterized by a strongly positive coordinate on the axis. These three variables all belong to the group of immune responses to pesticides in bees. Axis 2 contrasts two groups of strongly contributing variables. On one hand, the variables *Toll* and *spaetzle* are characterized by a strongly negative coordinate on the axis, and on the other hand, the variable VG is characterized by a strongly positive coordinate on the axis. With the exception of VG, which has the function of protecting bees against oxidative stress, the other two variables belong to immune response groups.

## 4 Discussion

From the obtained observations, we can state that imidacloprid downregulates the expression of *spaetzle* and zymosan mitigates this effect. In contrast, when amitraz is present with imidacloprid, the induction of gene expression by zymosan is not hindered by the presence of imidacloprid. We can infer that there is a competition between zymosan and imidacloprid on the signaling pathway that leads to the induction of *spaetzle* expression. Hence, imidacloprid may neutralize the effect of zymosan but amitraz seems to be antagonistic to imidacloprid and allows the induction of *spaetzle* by zymosan A even in the presence of imidacloprid in different imidacloprid-to-amitraz ratios. Our results for the decrease of *spaetzle* expression are in consensus with the laboratory application of imidacloprid on brown-eyed pupae (7), confirming the negative impact of imidacloprid on pathogen recognition by *spaetzle*.

In the context of immune activation, the effect of imidacloprid on *Toll* expression is dependent on the concentration, and though amitraz does not have an effect when hemocytes are immunologically challenged with zymosan, the combination of imidacloprid and amitraz show synergism regarding the decrease of the *Toll* expression. To add, imidacloprid and amitraz have similar downregulatory effects on *spaetzle* and *Toll* gene expression. We can also observe that the expressions of *spaetzle* and *Toll* are positively correlated. Comparing each treatment with zymosan absence (Zym 0) or presence (Zym 1), we can deduce that zymosan itself does not increase the expression of *Toll*, especially in the control treatments without pesticides, but instead, zymosan A attenuates the decrease of gene expression resulting from pesticide exposure. The results indicate that zymosan A has a regulatory role in maintaining the expression of the Toll receptor and limiting the negative effects on its production. Zymosan may have an antagonistic effect on imidacloprid and amitraz at this level. In addition, zymosan was not able to fully counter the effect of synergism between imidacloprid and amitraz mixtures.

Imidacloprid and amitraz affect the first levels of the Toll signaling pathway in a more intense manner than subsequent (intracellular) levels regarding the gene expression of Toll pathway components. In other words, imidacloprid and amitraz act on extracellular components of the signaling pathway, and the effect on intracellular components like *myD88* is less intense. This is also true for the observed synergism between the studied pesticides. The expression of *myd88* is more affected by amitraz than imidacloprid single exposures. This could show that the presence of an effect by imidacloprid on immune cells is dependent on the concentration of the pesticide. Intriguingly, the increase of *myD88* gene expression as a result of amitraz exposure was completely diminished when imidacloprid is co-present with amitraz. Thus, at the intracellular level, the interaction of imidacloprid and amitraz with hemocytes is antagonistic, unlike the extracellular level where strong synergism is detected. In contrast, zymosan appears to increase the gene expression of the intracellular *myD88* but has no effect by itself on the expression of *Toll* or *spaetzle*. This suggests a level-dependent inverted action by pesticides and zymosan on the Toll pathway.

In the IMD immune pathway, *relish* is a key component leading to the production of AMPs (29). Imidacloprid at 10 µg/ml significantly increases the expression of *relish* in contrast to amitraz single exposure. Again, the combination of amitraz and imidacloprid appears to have an antagonistic effect at this level where amitraz limits the increase of relish expression induced by 10 µg/ml imidacloprid. This is evident when amitraz counters the effect of 10 µg/ml imidacloprid in a concentration-dependent manner as observed in the pesticide mixture treatments. Note that concentration ratios between imidacloprid and amitraz may be very disruptive when concentrations are high as already observed. In laboratory conditions, the effect of amitraz on *relish* was strictly dependent on the developmental stage where the more developed the bee, the more the negative impact on *relish* (7). However, in the Zym 1 group, no treatment was significantly different from the zymosan control, implying that zymosan acts on sustaining the normal expression of *relish* in the IMD pathway. Fungal infections were already observed to activate the IMD pathway (51), but our results confirm that zymosan is also involved not just in the Toll pathway but also in the IMD pathway. Thus, the immune response to fungal infections comprises the activities of at least the Toll and IMD pathways referring to the complexity of the honeybee immune system specifically. The importance of MyD88 in the immune response was previously demonstrated in MyD88-deficient mice, which were unresponsive to stimulation by LPS (52) and loss of bacterial resistance (53). Thus, a decrease in Myd88 production caused by pesticide exposure may lead to immunosuppression, resulting in increased infection rate and ultimately risking the survivability of hives. However, a more comprehensive approach is needed for readouts for the immune pathways that can be further elucidated by quantifying other components of the immune response as melanization and the production of antimicrobial peptides.

As for *eater*, its production was reported to increase in honeybees when challenged with Varroa/DWS infection (49). Yet, in our study, immune activation did not increase the expression of *eater* compared to hemocytes that were exposed to zymosan with the exception of the 10I-50A treatment where zymosan exposure resulted in increased *eater* expression. Taking pesticide exposures into consideration, there was no change in gene expression compared to the respective controls except for pesticide mixtures 10I-50A and 50I-10A where a significant decrease in gene expression was observed compared to the control when not exposed to zymosan. However, when hemocytes were challenged with zymosan A, the expression of *eater* significantly increased with 10I-10A and 10I-50A when exposed to zymosan.

The two factors from the treatments that may have resulted in these observations could well be the high concentration of amitraz and zymosan. Hence, zymosan appears to alter the immuno-suppressive effect of 10I-50A into a stimulatory response regarding *eater*, though in all mixtures, phagocytosis itself was reduced in a preceding study (Sukkar et al., 2023 in review), suggesting that imidacloprid and amitraz affect phagocytosis by components other than *eater*.

Imidacloprid and amitraz appear to have no effect on the production of *vitellogenin (vg)* gene regardless of exposure to zymosan with the exception of 10I-10A (**Figure 2F**). This peculiar case infers that at given concentrations, imidacloprid and amitraz may act as reproductive disruptors when present simultaneously but not with single exposure or with mixtures of higher concentrations. A regulatory response may be activated to prevent alteration in *vg* levels. This could be connected to the observed trade-off between phagocytosis and the cytoprotective response. However, oral administration of sub-lethal doses of imidacloprid decreased vitellogenin expression levels in honeybees (49), suggesting that if there is an effect on vitellogenin from combinations and imidacloprid or amitraz, it may be more visible at the level of the whole organism or other types of cells. Variable degrees of vitellogenin expression were also reported between caged bees and bees in the field when exposed to 5 and 200 ppb imidacloprid from 1 to 2 days (50). In addition, PCA revealed a correlation between all the studied components of the Toll pathway. The expression of *spaetzle* and *Toll* was highly positively correlated and *myD88* was also positively correlated with both genes but to a lesser extent. This could refer back to the opposing effect of zymosan and pesticides depending on the level of the signaling pathway. The expression of *relish* and *eater* was also positively correlated with relish being closer to the genes of the Toll pathway.

These results demonstrate a competition of imidacloprid, amitraz, and zymosan A on the action of the immune pathways and immune elements. In addition, the effect of pesticides and zymosan is clearly evident on *Toll* and *spaetzle* followed by *myD88* and the Toll pathway, indicating that the risk of pesticide exposure affects the immune competence of honeybees starting from the early stages of infection where *Toll* and *spaetzle* are implicated in pathogen recognition and continues to downstream signaling.

This study provides insights into pesticide interactions specifically with hemocytes without interference by other components of the honeybee that may render the specific effect on hemocytes unobservable regarding gene expression analysis. The possible metabolism difference of imidacloprid and/or amitraz *in vivo* is not problematic when working with hemocytes since amitraz was already shown to have low metabolism in honeybees (17) and the used imidacloprid concentrations are higher than field levels. The latter is considered because hemocytes are not the main targets of imidacloprid and higher concentrations are needed to elucidate an effect. However, evaluating the effect of pesticides and pathogen interactions on partial components of honeybees and the whole organism is of equal importance since the end result is the main concern, but understanding the underlying effects on specific parts of the honeybee will provide a comprehensive view of the complexity that leads to the final observation on the organism level and the colony level.

## Data availability statement

The raw data supporting the conclusions of this article will be made available by the authors, without undue reservation.

## Ethics statement

Ethical review and approval was not required for the study on animals in accordance with the local legislation and institutional requirements.

## Author contributions

DS, PL-G, and JF-A conceived and designed the study. DS performed the experiments and wrote the first draft of the manuscript. PL-G and JF-A provided the funding. AK performed the statistical analysis and data treatment. DS, AK, PL-G, and JF-A read, revised, and approved the manuscript for submission. All authors contributed to the article and approved the submitted version.

## References

[1] vanEngelsdorp D Evans JD Saegerman C Mullin C Haubruge E Nguyen BK. Colony collapse disorder: A descriptive study. PLoS One (2009) 4(8). doi: 10.1371/journal.pone.0006481 PMC2715894 19649264

[2] Ellis JD Evans JD Pettis J. Colony losses, managed colony population decline, and Colony Collapse Disorder in the United States. J Apicultural Res (2010) 49:134–6. doi: 10.3896/IBRA.1.49.1.30

[3] Cox-Foster DL Conlan S Holmes EC Palacios G Evans JD Moran NA. A metagenomic survey of microbes in honey bee colony collapse disorder. Sci (1979) (2007) 318(5848):283–7. doi: 10.1126/science.1146498 17823314

[4] Galajda R Valenčáková A Sučik M Kandráčová P. Nosema disease of European honey bees. J Fungi (2021) 7(9):714. doi: 10.3390/jof7090714 PMC8468538 34575752

[5] Higes M Martín-Hernández R Botías C Bailón EG González-Porto AV Barrios L. How natural infection by Nosema ceranae causes honeybee colony collapse. Environ Microbiol (2008) 10(10):2659–69. doi: 10.1111/j.1462-2920.2008.01687.x 18647336

[6] Paxton RJ. Does infection by Nosema ceranae cause “colony Collapse Disorder” in honey bees (Apis mellifera)? J Apic Res (2010) 49(1):80–4. doi: 10.3896/IBRA.1.49.1.11

[7] Tesovnik T Zorc M Gregorc A Rinehart T Adamczyk J Narat M. Immune gene expression in developing honey bees (Apis mellifera L.) simultaneously exposed to imidacloprid and Varroa destructor in laboratory conditions. J Apic Res (2019) 58(5):730–9. doi: 10.1371/journal.pone.0187079

[8] Pettis JS Vanengelsdorp D Johnson J Dively G. Pesticide exposure in honey bees results in increased levels of the gut pathogen Nosema. Naturwissenschaften (2012) 99(2):153–8. doi: 10.1007/s00114-011-0881-1 PMC3264871 22246149

[9] Paris L Peghaire E Moné A Diogon M Debroas D Delbac F. Honeybee gut microbiota dysbiosis in pesticide/parasite co-exposures is mainly induced by Nosema ceranae. J Invertebr Pathol (2020) 172:107348. doi: 10.1016/j.jip.2020.107348 32119953

[10] Alberoni D Di Gioia D Baffoni L. Alterations in the microbiota of caged honeybees in the presence of Nosema ceranae infection and related changes in functionality. Microb Ecol (2023) 86(1):601–16. doi: 10.1007/s00248-022-02050-4 PMC10293464 35819480

[11] Rouzé R Moné A Delbac F Belzunces L Blot N. The Honeybee Gut Microbiota Is Altered after Chronic Exposure to Different Families of Insecticides and Infection by Nosema ceranae. Microbes Environ (2019) 34(3):226–33. doi: 10.1264/jsme2.ME18169 PMC6759349 31378758

[12] Paris L El Alaoui H Delbac F Diogon M. Effects of the gut parasite Nosema ceranae on honey bee physiology and behavior. Curr Opin Insect Sci (2018) 26:149–54. doi: 10.1016/j.cois.2018.02.017 29764655

[13] Jeschke P Nauen R Schindler M Elbert A. Overview of the status and global strategy for neonicotinoids. J Agric Food Chem (2011) 59(7):2897–908. doi: 10.1021/jf101303g 20565065

[14] Mitchell EAD Mulhauser B Mulot M Mutabazi A Glauser G Aebi A. A worldwide survey of neonicotinoids in honey. Science (1979) 358(6359):109–11. doi: 10.1126/science.aan3684 28983052

[15] Rosenkranz P Aumeier P Ziegelmann B. Biology and control of Varroa destructor. J Invertebr Pathol (2010) 103 Suppl 1(SUPPL. 1):S96–S119. doi: 10.1016/j.jip.2009.07.016 19909970

[16] Ostiguy N Drummond FA Aronstein K Eitzer B Ellis JD Spivak M. Honey bee exposure to pesticides: A four-year nationwide study. Insects (2019) 10(1):1–34. doi: 10.3390/insects10010013 PMC6359572 30626027

[17] Guo L Fan X Qiao X Montell C Huang J. An octopamine receptor confers selective toxicity of amitraz on honeybees and Varroa mites. Elife (2021) 10. doi: 10.7554/eLife.68268.sa2 PMC8313232 34263722

[18] Dai P Jack CJ Mortensen AN Bustamante TA Ellis JD. Chronic toxicity of amitraz, coumaphos and fluvalinate to Apis mellifera L. larvae reared in vitro. Sci Rep (2018) 8(1). doi: 10.1038/s41598-018-24045-3 PMC5884784 29618776

[19] O’Neal ST Brewster CC Bloomquist JR Anderson TD. Amitraz and its metabolite modulate honey bee cardiac function and tolerance to viral infection. J Invertebr Pathol (2017) 149:119–26. doi: 10.1016/j.jip.2017.08.005 28797906

[20] Feldhaar H Gross R. Immune reactions of insects on bacterial pathogens and mutualists. Microbes Infect (2008) 10(9):1082–8. doi: 10.1016/j.micinf.2008.07.010 18672091

[21] Larsen A Reynaldi F Guzman-Novoa E. Fundaments of the honey bee ( Apis mellifera ) immune system. Review (2019) 37(3):521–33. doi: 10.1016/j.cvfa.2021.06.007

[22] Melcarne C Lemaitre B Kurant E. Phagocytosis in Drosophila: From molecules and cellular machinery to physiology. Insect Biochem Mol Biol (2019) 109:1–12. doi: 10.1016/j.ibmb.2019.04.002 30953686

[23] Kocks C Cho JH Nehme N Ulvila J Pearson AM Meister M. Eater, a transmembrane protein mediating phagocytosis of bacterial pathogens in drosophila. Cell (2005) 123(2):335–46. doi: 10.1016/j.cell.2005.08.034 16239149

[24] Bretscher AJ Honti V Binggeli O Burri O Poidevin M Kurucz É. The Nimrod transmembrane receptor Eater is required for hemocyte attachment to the sessile compartment in Drosophila melanogaster. Biol Open (2015) 4(3):355–63. doi: 10.1242/bio.201410595 PMC4359741 25681394

[25] Kawasaki T Kawai T. Toll-like receptor signaling pathways. Front Immunol (2014) 5(SEP):1–8. doi: 10.3389/fimmu.2014.00461 25309543 PMC4174766

[26] Sadeghalvad M Mohammadi-Motlagh HR Rezaei N. Toll-like receptors. Encyclopedia Infection Immun (2022) 1:130–43. doi: 10.1016/B978-0-12-818731-9.00044-6

[27] Valanne S Wang JH Rämet M. The Drosophila Toll signaling pathway. J Immunol (2011) 186(2):649–56. doi: 10.4049/jimmunol.1002302 21209287

[28] Anderson KV. Toll signaling pathways in the innate immune response. Curr Opin Immunol (2000) 12(1):13–9. doi: 10.1016/S0952-7915(99)00045-X 10679407

[29] Evans JD Aronstein K Chen YP Hetru C Imler JL Jiang H. Immune pathways and defence mechanisms in honey bees Apis mellifera. Insect Mol Biol (2006) 15(5):645–56. doi: 10.1111/j.1365-2583.2006.00682.x PMC1847501 17069638

[30] Mogensen TH. Pathogen recognition and inflammatory signaling in innate immune defenses. Clin Microbiol Rev (2009) 22(2):240–73. doi: 10.1128/CMR.00046-08 PMC2668232 19366914

[31] Murphy K Kenneth M Weaver C Janeway C. Janeway’s immunobiology. 9th ed. New York, USA: New York, NY: Garland Science (2017).

[32] Stothers CL Burelbach KR Owen AM Patil NK McBride MA Bohannon JK. β-glucan induces distinct and protective innate immune memory in differentiated macrophages. J Immunol (2021) 207(11):2785–98. doi: 10.4049/jimmunol.2100107 PMC8612974 34740960

[33] Aymeric JL Givaudan A Duvic B. Imd pathway is involved in the interaction of Drosophila melanogaster with the entomopathogenic bacteria, Xenorhabdus nematophila and Photorhabdus luminescens. Mol Immunol (2010) 47(14):2342–8. doi: 10.1016/j.molimm.2010.05.012 20627393

[34] Weber ANR Tauszig-Delamasure S Hoffmann JA Lelièvre E Gascan H Ray KP. Binding of the Drosophila cytokine Spätzle to Toll is direct and establishes signaling. Nat Immunol (2003) 4(8):794–800. doi: 10.1038/ni955 12872120

[35] Brutscher LM Daughenbaugh KF Flenniken ML. Antiviral defense mechanisms in honey bees. Curr Opin Insect Sci (2015) 10(Ccd):71–82. doi: 10.1016/j.cois.2015.04.016 26273564 PMC4530548

[36] Marr KA Balajee SA Hawn TR Ozinsky A Pham U Akira S. Differential role of MyD88 in macrophage-mediated responses to opportunistic fungal pathogens. Infect Immun (2003) 71(9):5280–6. doi: 10.1128/IAI.71.9.5280-5286.2003 PMC187297 12933875

[37] Benjamin SJ Hawley KL Vera-Licona P La Vake CJ Cervantes JL Ruan Y. Macrophage mediated recognition and clearance of Borrelia burgdorferi elicits MyD88-dependent and -independent phagosomal signals that contribute to phagocytosis and inflammation. BMC Immunol (2021) 22(1):1–16. doi: 10.1186/s12865-021-00418-8 34000990 PMC8127205

[38] Kornstädt L Pierre S Weigert A Ebersberger S Schäufele TJ Kolbinger A. Bacterial and fungal toll-like receptor activation elicits type I IFN responses in mast cells. Front Immunol (2021) 12:11. doi: 10.3389/fimmu.2020.607048 PMC7907501 33643293

[39] Jiang LI Sternweis PC Wang JE. Zymosan activates protein kinase A *via* adenylyl cyclase VII to modulate innate immune responses during inflammation. Mol Immunol (2013) 54(1):14–22. doi: 10.1016/j.molimm.2012.10.027 23178822 PMC3548999

[40] Sato M Sano H Iwaki D Kudo K Konishi M Takahashi H. Direct binding of toll-like receptor 2 to Zymosan, and Zymosan-induced NF-κB activation and TNF-α Secretion are down-regulated by lung collectin surfactant protein A. J Immunol (2003) 171(1):417–25. doi: 10.4049/jimmunol.171.1.417 12817025

[41] Underhill DM. Macrophage recognition of zymosan particles. J Endotoxin Res (2003) 9(3):176–80. doi: 10.1177/09680519030090030601 12831459

[42] Arrese EL Soulages JL. Insect fat body: energy, metabolism, and regulation. Annu Rev Entomol (2010) 55:207–25. doi: 10.1146/annurev-ento-112408-085356 PMC3075550 19725772

[43] Salmela H Amdam GV Freitak D. Transfer of immunity from mother to offspring is mediated *via* egg-yolk protein vitellogenin. PLoS Pathog (2015) 11(7). doi: 10.1371/journal.ppat.1005015 PMC4521805 26230630

[44] Harwood G Salmela H Freitak D Amdam G. Social immunity in honey bees: royal jelly as a vehicle in transferring bacterial pathogen fragments between nestmates. J Exp Biol (2021) 224(7). doi: 10.1242/jeb.231076 34424968

[45] Seehuus SC Norberg K Gimsa U Krekling T Amdam GV. Reproductive protein protects functionally sterile honey bee workers from oxidative stress. Proc Natl Acad Sci U S A (2006) 103(4):962–7. doi: 10.1073/pnas.0502681103 PMC1347965 16418279

[46] Hunter WB. Medium for development of bee cell cultures (Apis mellifera: Hymenoptera: Apidae). In Vitro Cell Dev Biol Anim (2010) 46(2):83–6. doi: 10.1007/s11626-009-9246-x 20033792

[47] Simone M Evans JD Spivak M. Resin collection and social immunity in honey bees. Evolution (2009) 63(11):3016–22. doi: 10.1111/j.1558-5646.2009.00772.x 19619221

[48] Lourenço AP Mackert A Cristino ADS Simões ZLP. Validation of reference genes for gene expression studies in the honey bee, Apis mellifera, by quantitative real-time RT-PCR. Apidologie (2008) 39(3):372–85. doi: 10.1051/apido:2008015

[49] Abbo PM Kawasaki JK Hamilton M Cook SC DeGrandi-Hoffman G Li WF. Effects of Imidacloprid and Varroa destructor on survival and health of European honey bees, Apis mellifera. Insect Sci (2017) 24(3):467–77. doi: 10.1111/1744-7917.12335 26990560

[50] De Smet L Hatjina F Ioannidis P Hamamtzoglou A Schoonvaere K Francis F. Stress indicator gene expression profiles, colony dynamics and tissue development of honey bees exposed to sub-lethal doses of imidacloprid in laboratory and field experiments. PLoS One (2017) 12(2):e0171529. doi: 10.1371/journal.pone.0171529 28182641 PMC5300173

[51] Ramirez JL Muturi EJ Barletta ABF Rooney AP. The Aedes aEgypti IMD pathway is a critical component of the mosquito antifungal immune response. Dev Comp Immunol (2019) 95:1–9. doi: 10.1016/j.dci.2018.12.010 30582948

[52] Kawai T Adachi O Ogawa T Takeda K Akira S. Unresponsiveness of myD88-deficient mice to endotoxin. Immunity (1999) 11(1):115–22. doi: 10.1016/S1074-7613(00)80086-2 10435584

[53] Scanga CA Bafica A Feng CG Cheever AW Hieny S Sher A. MyD88-deficient mice display a profound loss in resistance to mycobacterium tuberculosis associated with partially impaired th1 cytokine and nitric oxide synthase 2 expression. Infect Immun (2004) 72(4):2400. doi: 10.1128/IAI.72.4.2400-2404.2004 15039368 PMC375220

