# Corrigendum: A clash on the Toll pathway: competitive action between pesticides and zymosan A on components of innate immunity in *Apis mellifera*

**DOI:** 10.3389/fimmu.2023.1294963

**Published:** 2023-09-25

**Authors:** Dani Sukkar, Ali Kanso, Philippe Laval-Gilly, Jairo Falla-Angel

**Affiliations:** ^1^ Université de Lorraine, INRAE, LSE, F-54000 Nancy, France; ^2^ Biology Department, Faculty of Sciences I, Lebanese University, Hadath, Lebanon

**Keywords:** Toll pathway, immune modulation, β-glucan, imidacloprid, amitraz, honeybees, immune genes

In the published article, there was an error in affiliation 1. Instead of “Laboratoire Sols et Environnement, Institut National de Recherche pour l’Agriculture, l’Alimentation et l’Environnement (INRAE), Université de Lorraine, Nancy, France”, it should be “Université de Lorraine, INRAE, LSE, F-54000 Nancy, France”.

In the published article, there was an error in the published article.

A correction has been made to the **Acknowledgments**. This section previously stated:

“We like to thank Dr. Michel Aillerie and Sandhya Malladi for their aid in maintaining the bee hives and Antoine Bonnefoy for technical support.”

The corrected section appears below:

“We like to thank Dr. Michel Aillerie and Sandhya Malladi for their aid in maintaining the beehives and Antoine Bonnefoy for technical support. We also thank IUT de Thionville-Yutz (Université de Lorraine) and its Plateforme de Recherche, Transfert de Technologie et Innovation (PRTI) for providing the platform and equipment to perform the experiments.”

The authors apologize for these errors and state that they do not change the scientific conclusions of the article in any way. The original article has been updated.

